# Evaluation of 3D vertebral and pelvic position by surface topography in asymptomatic females: presentation of normative reference data

**DOI:** 10.1186/s13018-021-02843-2

**Published:** 2021-12-04

**Authors:** Claudia Wolf, Ulrich Betz, Janine Huthwelker, Jürgen Konradi, Ruben Sebastian Westphal, Meghan Cerpa, Lawrence Lenke, Philipp Drees

**Affiliations:** 1 Institute of Physical Therapy, Prevention and Rehabilitation, University Medical Center of the Johannes Gutenberg University Mainz, Langenbeckstrasse 1, 55131 Mainz, Germany; 2 Institute of Medical Biostatistics, Epidemiology and Informatics, University Medical Center of the Johannes Gutenberg University Mainz, Obere Zahlbacher Straße 69, 55131 Mainz, Germany; 3 Department of Orthopedic Surgery, Columbia University Medical Center, 5141 Broadway, New York, NY 10034 USA; 4 Department of Orthopedics and Trauma Surgery, University Medical Center of the Johannes Gutenberg University Mainz, Langenbeckstrasse 1, 55131 Mainz, Germany

**Keywords:** Rasterstereography, Stance, Reference data, Healthy women, Asymmetrical posture

## Abstract

**Background:**

Deviations from a conventional physiologic posture are often a cause of complaint. According to current literature, the upright physiological spine posture exhibits inclinations in the sagittal plane but not in the coronal and transverse planes, but individual vertebral body positions of asymptomatic adults have rarely been described using surface topography. Therefore, this work aims to form a normative reference dataset for the thoracic and lumbar vertebral bodies and for the pelvis in all three planes in asymptomatic women.

**Methods:**

In a prospective, cross-sectional, monocentric study, 100 pain-free asymptomatic women, aged 20–64 years were enrolled. Habitual standing positions of the trunk were measured using surface topography. Data were analyzed in all three planes. Age sub-analysis was: 1) ages ≤ 40 years and 2) ages ≥ 41 years. Two-sample t-tests were used for age comparisons of the vertebral bodies, vertebra prominence (VP)–L4, and global parameters. One-sample t-tests were used to test deviations from symmetrical zero positions of VP–L4.

**Results:**

Coronal plane: on average, the vertebral bodies were tilted to the right between the VP and T4 (maximum: T2 − 1.8° ± 3.2), while between T6 and T11 they were tilted to the left (maximum: T7 1.1° ± 1.9). T5 and L2 were in a neutral position, overall depicting a mean right-sided lateral flexion from T2 to T7 (apex at T5). Sagittal plane: the kyphotic apex resided at T8 with − 0.5° ± 3.6 and the lumbar lordotic apex at L3 with − 2.1° ± 7.4. Transverse plane: participants had a mean vertebral body rotation to the right ranging from T6 to L4 (maximum: T11 − 2.2° ± 3.5). Age-specific differences were seen in the sagittal plane and had little effect on overall posture.

**Conclusions:**

Asymptomatic female volunteers standing in a habitual posture displayed an average vertebral rotation and lateral flexion to the right in vertebral segments T2–T7. The physiological asymmetrical posture of women could be considered in spinal therapies. With regard to spinal surgery, it should be clarified whether an approximation to an absolutely symmetrical posture is desirable from a biomechanical point of view? This data set can also be used as a reference in clinical practice.

*Trial registration:* This study was registered with WHO (INT: DRKS00010834) and approved by the responsible ethics committee at the Rhineland–Palatinate Medical Association (837.194.16).

**Supplementary Information:**

The online version contains supplementary material available at 10.1186/s13018-021-02843-2.

## Background

Deviations from a conventional physiologic posture often cause pathologic complaints due to increased stress on the musculoskeletal system. Technological-based posture analyses are not commonly performed. While some centers have an upright-open MRI [1], this technique is not always financially feasible and often is not suitable for a wide range of use. Conversely, using surface topography (ST) we can estimate vertebral positions and describe physiologic posture with high precision. ST is an established and reliable technique used to analyze the surfaces of a patient’s body while in an upright standing position [2–9].

To systematically assess individual posture measurements, it is necessary to have a normative dataset for comparison. Recent literature offers limited data describing the relationship of vertebral body positions and upright physiologic posture of asymptomatic adults [10–14]. Additionally, the literature only describes global spinal relationships, for example, thoracic kyphosis (TK) and lumbar lordosis (LL), but is lacking detailed data on individual vertebral segments. Currently, this technology is mainly used in scoliotic patients, who are predominantly female. Our study reports the multi-planar positions of individual vertebral bodies and global spinal parameters in a normatively asymptomatic female population. This is essential to understand the nature of spinal pathologies and deformities.

## Methods

This study is a sub-analysis of data from a cohort of 201 asymptomatic participants assessing posture and gait while in a habitual stance. For data related to gait analysis, refer to Betz et al. [15, 16]. This study was registered with WHO (INT: DRKS00010834) and approved by the responsible ethics committee at the Rhineland–Palatinate Medical Association (837.194.16).

### Participants

This was a prospective, cross-sectional, monocentric study. Healthy participants, aged 18–70 years, were recruited through media advertising announcements and gave informed consent prior to inclusion. Participants were excluded in the event of abnormal sensory or motor test results (e.g., timed "Up & Go" [17], Two-Minute Walk Test [18], Back Performance Scale [19], Range of Motion Test [20], and the International Physical Activity Questionnaire [21]), amputation of the lower extremities, obesity (Body Mass Index - BMI > 30), or previous orthopedic surgery in the thoracic or lumbar spine and/or pelvis (e.g., nucleotomy). In detail, the participants had no pain, neither in the spine nor in the extremities; they had no need for therapy in the extremities due to pain or dysfunction in the 6 months prior to the study (in the spine 12 months); there was the necessary mobility in the extremities and the spine for a natural gait (hip joint: flexion/extension: > 25/0/20, abduction/adduction: > 5/0/10, internal/external rotation: > 5/0/10; knee joint: flexion/extension: > 60/0/0; upper ankle joint: dorsal extension/ plantar flexion: > 10/0/15; shoulder joint: flexion/extension: > 25/0/20; elbow joint: flexion/extension: > 45/20/0; spine: frontal plane right/left: > 10/0/10, transversal plane right/left: > 10/0/10); 100 pain-free healthy women aged 20–64 years were enrolled and then stratified by age into two groups (≤ 40 years: younger group (YG); > 40 years: older group (OG)).

### Measuring technique

The DIERS Formetric III 4D™ system (DICAM v3.7.1.7; DIERS International GmbH, Schlangenbad, Germany), a light-optical scanning method based on ST was used for this study. The three-dimensional (3D) camera unit records a defined position with a frequency up to 60 Hz. Reconstructed skeletal structures were processed by the system [spine between the seventh cervical (C) and the fourth lumbar (L) vertebral bodies and the iliosacral joints]. The spatial, individual three-dimensional position is then calculated for each vertebra and the pelvis [22–24].

### Data analysis

Central landmarks were palpated and marked by the same person with skin-compatible markers to obtain measurements. Markers were placed at: outer edge of both acromia; processus spinosus vertebrae cervicalis VII [different from vertebra prominens (VP)]; both spina ilica posterior superior (SIPS) [comparable with left and right dimple], and the processus spinosus vertebrae thoracicae III and XII.

Participants stood in an upright habitual stance, eyes looking at a point 20 cm below each body height, for 12 separate recordings. An average across all 12 measures was taken. We chose a subset of 16 typical global and all 51 specific parameters in order to describe the spines of healthy women. An additional file 1 explains the individual parameters in detail and defines them (see Additional file 1). Global parameters (e.g., TK-angle or maximum surface rotation) are frequently associated with the topography of the surface. Specific parameters, especially the rotation of vertebral bodies, happen in situ; therefore, the direction of rotation changes. During stance measurements the DIERS software calculates each parameter relative to the neutral pelvic rotation thereby re-defining the transversal plane, excluding the pelvis (see Additional file 1).

**Fig. 1:**
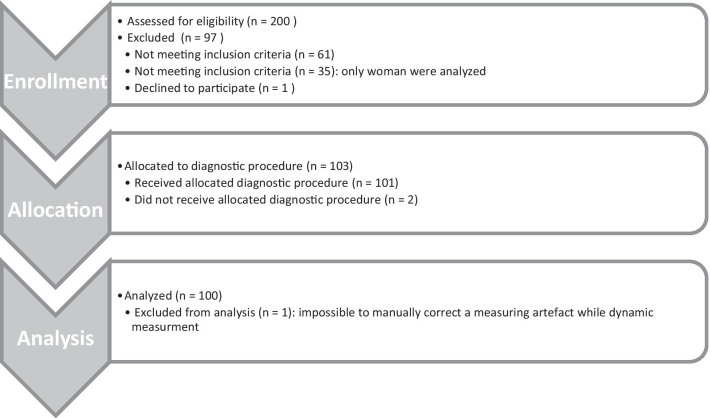
Transparent reporting of trial adopted from CONSORT

After a systematic review of measurement artifacts, a data set was removed. We exported the raw data with a DIERS application (DICAM v3.5.0beta11; DIERS International GmbH, Schlangenbad, Germany). These files were combined via Statistical Analysis System (SAS v9.4). Statistical Package for the Social Sciences (SPSS v23) and Microsoft Excel (Version 2010) were used for statistical analysis. The process of enrollment, allocation, and analysis is summarized in Fig. 1.

## Results

First, the statistical procedure and the most relevant results of the age and BMI are described. Secondly, the parameters are reported along with the variable relationships and associations.

### Participants

The mean ±  standard deviation (SD) age of the entire group (EG) was 39.8 ± 12.1 (20–64) years with a BMI of 23.0 ± 2.9 kg/m². The mean age and BMI of the YG were 28.9 ± 5.0 (20–40) and 22.3 ± 2.7 kg/m², and the OG was 50.7 ± 5.3 (42–64), 23.7 ± 3.0 kg/m².

In the coronal (CP) and transverse planes (TP) for the EG, we expected the vertebral bodies in an average spine posture to be in symmetrical zero positions. Comparatively, due to the natural TK and LL curvature of the spine in the sagittal plane (SP) values were expected not to equal zero. One-sample t-tests (significance level p < 0.05) and effect sizes according to Cohen’s d (d = abs[(means–0)/SD])[25] were calculated (Tables 1, 2).

**Table 1:** Specific parameters in coronal, sagittal, and transversal plane, of entire, younger, and older group

Specific parameters	VP	T1	T2	T3	T4	T5	T6	T7	T8	T9	T10	T11	T12	L1	L2	L3	L4
*Coronal plane: vertebral lateral flexion (°)*																	
EG	− 0.9 ± 2.5	− 1.4 ± 2.7	− 1.8 ± 3.2	− 1.5 ± 3.3	− 0.8 ± 3.1	0.0 ± 2.6	0.8 ± 2.0	1.1 ± 1.8	1.1 ± 2.0	0.9 ± 2.0	0.7 ± 1.9	0.6 ± 2.0	0.6 ± 2.1	0.3 ± 1.8	0.1 ± 1.9	0.3 ± 2.7	0.8 ± 3.3
EG vs. 0	.000*	.000*	.000*	.000*	.012*	.861	.000*	.000*	.000*	.000*	.001*	.003*	.009*	.084	.698	.309	.014*
t-value	− 3.7	− 5.0	− 5.6	− 4.5	− 2.5	0.2	3.9	6.2	5.6	4.4	3.5	3.1	2.7	1.7	0.4	1.0	2.5
Cohen’s d	0.4	0.5	0.6	0.5	0.3	0.0	0.4	0.6	0.6	0.4	0.40	0.3	0.3	0.2	0.0	0.1	0.2
YG	− 0.7 ± 2.2	− 1.0 ± 2.5	− 1.3 ± 2.9	− 0.9 ± 3.0	− 0.2 ± 3.0	0.4 ± 2.6	1.0 ± 2.0	1.1 ± 1.9	1.0 ± 1.9	0.9 ± 1.8	0.8 ± 1.7	0.8 ± 2.0	0.5 ± 2.0	0.0 ± 1.4	− 0.3 ± 1.6	0.1 ± 2.6	0.9 ± 3.1
OG	− 1.2 ± 2.7	− 1.7 ± 3.0	− 2.3 ± 3.4	− 2.1 ± 3.4	− 1.4 ± 3.2	− 0.4 ± 2.6	0.6 ± 2.0	1.2 ± 1.8	1.2 ± 2.1	0.9 ± 2.2	0.5 ± 2.1	0.5 ± 2.0	0.6 ± 2.2	0.6 ± 2.1	0.4 ± 2.0	− 0.3 ± 2.9	− 0.8v3.6
YG vs. OG	.343	.213	.099	.062	.076	.124	.393	.738	.580	.999	.485	.456	.841	.105	.045*	.456	.903
*Sagittal plane: vertebral flexion/extension (°)*																	
EG	29.6 ± 6.6	27.5 ± 6.3	21.6 ± 6.2	15.2 ± 5.0	11.2 ± 4.3	8.7 ± 4.1	6.3 ± 3.8	3.7 ± 3.7	− 0.5 ± 3.6	− 6.1 ± 3.7	− 11.5 ± 4.0	− 16.1 ± 4.6	− 18.8 ± 5. 6	− 18.1 ± 5.9	− 13.6 ± 6.1	− 2.2 ± 7.4	13.2 ± 6.5
EG vs. 0;	.000*	.000*	.000*	.000*	.000*	.000*	.000*	.000*	.159	.000*	.000*	.000*	.000*	.000*	.000*	.004*	.000*
t-value	44.9	43.5	35.0	30.4	26.3	21.3	16.5	9.9	− 1.4	− 16.4	− 28.5	− 35.0	− 33.8	− 30.7	− 22.3	− 2.9	20.1
Cohen’s d	4.5	4.4	3.5	3.0	2.6	2.1	1.7	1.0	0.1	1.6	2.9	3.5	3.4	3.1	2.2	0.3	2.0
YG	27.2 ± 5.3	25.7 ± 5.5	20.8 ± 6.1	14. 8 ± 5.1	10.8 ± 4.2	8.2 ± 3.9	6.1 ± 3.5	3.6 ± 3.5	− 0.4 ± 3.5	− 5.7 ± 3.6	− 11.0 ± 3.9	− 15.3 ± 4.6	− 17.6 ± 5.7	− 16.8 ± 5.9	− 12.8 ± 6.3	− 1.5 ± 7.4	12.7 ± 6.7
OG	32.1 ± 6.9	29.4 ± 6.6	22.5 ± 6.2	15.6 ± 4.9	11.6 ± 4.3	9.1 ± 4.3	6.6 ± 4.1	3.7 ± 3.9	− 0.7 ± 3.6	− 6.4 ± 3.9	− 12.0 ± 4.1	− 17.0 ± 4.5	− 20.0 ± 5.2	− 19.4 ± 5.6	− 4.4 ± 5.8	− .8 ± 7.4	13.6 ± 6.4
YG vs. OG	.000*	.002*	.161	.422	.331	.249	.465	.808	.664	.324	.205	.052	.028*	.025*	.168	.376	.504
*Transversal plane: vertebral rotation (°)*																	
EG	0.0 ± 0.2	0.18 ± 0.4	0.1 ± 0.7	0.2 ± 1.3	0.2 ± 2.2	0.0 ± 3.1	− 0.4 ± 3.7	− 1.0 ± 3.9	− 1.6 ± 3.8	− 1.9 ± 3.7	− 2.2 ± 3.5	− 2.2 ± 3.5	− 2.2 ± 3.5	− 2.0 ± 3.5	− 1.5 ± 3.2	− 0.8 ± 2.4	− 0.3 ± 1.4
EG vs. 0	.068	.040*	.054	.141	.401	.990	.244	.011*	.000*	.000*	.000*	.000*	.000*	.000*	.000*	.001*	.043*
t-value	1.8	2.1	2.0	1.5	0.8	0.0	− 1.2	− 2.6	− 4.1	− 5.3	− 6.1	− 6.4	− 6.3	− 5.7	− 4.6	− 3.3	− 2.0
Cohen’s d	0.2	0.2	0.2	0.1	0.1	0.0	0.1	0.3	0.4	0.5	0.6	0.6	0.6	0.6	0.5	0.3	0.2
YG	0.3 ± 0.2	0.0 ± 0.4	0.0 ± 0.6	0.0 ± 1.1	− 0.1 ± 1.9	− 0.4 ± 2.8	− 0.9 ± 3.4	− 1.4 ± 3.6	− 1.9 ± 3.7	− 2.2 ± 3.6	− 2.4 ± 3.4	− 2.5 ± 3.1	− 2.5 ± 3.0	− 2.2 ± 3.1	− 1.6 ± 2.9	− 0.7 ± 2.2	− 0.2 ± 1.3
OG	0.1 ± 0.2	0.1 ± 0.4	0.2 ± 0.8	0.4 ± 1.5	0.5 ± 2.4	0.4 ± 3.4	0.0 ± 4.0	− 0.6 ± 4.1	− 1.2 ± 3.9	− 1.7 ± 3.8	− 1.9 ± 3.7	− 1.9 ± 3.8	− 1.8 ± 3.9	− 1.7 ± 3.8	− 1.4 ± 3.5	− 0.9 ± 2.6	− 0.4 ± 1.5
YG vs. OG	.498	.219	.164	.186	.193	.204	.217	.278	.393	.474	.456	.355	.321	.460	.797	.727	.431

Specific parameters in coronal, sagittal, and transversal plane (mean ± SD) of entire group (EG) and their p-, t-values (df = 99) and Cohen’s d; *: p < 0.05; parameters of younger (YG) and older group (OG) and their p-values

**Table 2:** Global parameters and literature review comparison in three planes of entire, younger, and older group

Coronal plane	Trunk imbalance (VP–DM)		Maximum apical deviation		Shoulder obliquity		Pelvic obliquity	
	(mm)	(°)	(mm) (+ max)	(mm) (− max)	(mm)	(°)	(mm)	(°)
EG; n = 100	− 1.9 ± 8.9	− 0.2 ± 1.1	2.9 ± 2.8	− 5.1 ± 3.7	− 8.2 ± 9.6	− 1.2 ± 1.5	− 0.2 ± 2.2	− 0.1 ± 1.2
EG vs. 0	.033*	.033*	.000*	.000*	.000*	.000*	.412	.394
t-value (df = 99)	− 2.2	− 2.2	10.4	− 14.0	− 8.6	− 8.6	− 0.8	− 0.9
Cohen’s d	0.2	0.2	1.0	1.4	0.9	0.9	0.1	0.1
YG	− 2.6 ± 7.5	− 0.3 ± 0.9	3.2 ± 3.0	− 4.4 ± 3.6	− 7.3 ± 8.9	− 1.1 ± 1.3	− 0.2 ± 2.2	− 0.1 ± 1.2
OG	− 1.2 ± 10.2	− 0.2 ± 1.2	2.6 ± 2.6	− 5.8 ± 3.7	− 9.2 ± 10.2	− 1.4 ± 1.6	− 1.2 ± 2.1	− 0.1 ± 1.1
YG vs. OG	.422	.463	.303	.058	.346	.343	.929	.916
*Literature comparison*								
Degenhardt, et al. [10]	1.0 ± 7.2	0.1 ± 0.8	7.9 ± 5.8 ‡	− 5.0 ± 4.1	n.v.	n.v.	0.2 ± 5.9	0.0 ± 3.5
Degenhardt, et al. [11]	1.3 ± 5.6	0.2 ± 0.7	8.0 ± 5.1	− 4.6 ± 2.9	n.v.	n.v.	− 0.1 ± 5.1	− 0.2 ± 2.9
Schröder et al. [12]	6.9 ± 4.6	n.v.	n.v.	n.v.	n.v.	n.v.	3.1 ± 2.5	n.v.
Hamm [13], Michalik et al. [14]	n.v.	− 0.1 ± 0.9	n.v.	n.v.	n.v.	n.v.	n.v.	− 0.4 ± 2.8

Sagittal plane	Trunk inclination (VP–DM)		Thoracic kyphosis (ICT–ITL)	Lumbar lordosis (ITL–ILS)	Pelvic inclination (dimples)
	(mm)	(°)	(°)	(°)	(°)
EG; n = 100	25.4 ± 17.2	3.1 ± 2.1	47.3 ± 8.5	43.8 ± 9.1	18.7 ± 9.0
EG vs. 0	.000*	.000*	.000*	.000*	.000*
t-value (df = 99)	14.8	14.8	55.8	47.9	20.8
Cohen’s d	1.5	1.5	5.6	4.8	2.1
YG	25.7 ± 16.9	3.2 ± 2.1	44.2 ± 7.9	41.5 ± 9.2	20.5 ± 8.0
OG	25.1 ± 17.6	3.0 ± 2.1	50.4 ± 7.9	46.1 ± 8.6	16.9 ± 9.6
YG vs. OG	.874	.782	.000*	.011*	.042*
*Literature comparison*					
Degenhardt, et al. [10]	26.0 ± 18.7	3.1 ± 2.3	48.1 ± 9.1	35.6 ± 8.4	17.9 ± 6.0
Degenhardt, et al. [11]	n.v.	n.v.	48.5 ± 8.3	35.4 ± 7.6	19.7 ± 7.3 (symm. line)
Schröder et al. [12]	12.3 ± 17.9	n.v.	47.1 ± 8.6	42.7 ± 8.2	21.9 ± 4.8 ^†^
Hamm [13], Michalik et al. [14]	n.v.	2.1 ± 2.4	44.0 ± 8.6 (VP–T12)	37.4 ± 9.8 (T12–DM)	n.v.

Transversal plane	Maximum surface rotation		Pelvic rotation
	(°) (+ max)	(°) (− max)	(°)
EG; n = 100	2.0 ± 2.4	− 3.9 ± 2.7	− 0.1 ± 0.6
EG vs. 0	.000*	.000*	.244
t-value (df = 99)	8.2	− 14.6	− 1.2
Cohen’s d	0.8	1.5	0.1
YG	1.7 ± 1.9	− 3.9 ± 2.8	− 0.1 ± 0.6
OG	2.3 ± 2.8	− 3.9 ± 2.6	− 0.1 ± 0.6
YG vs. OG	.246	.957	.922
*Literature comparison*			
Degenhardt, et al. [10]	5.6 ± 3.4 ^‡^	− 4.6 ± 2.9	− 0.3 ± 2.8
Degenhardt, et al. [11]	5.7 ± 2.8	− 4.5 ± 2.4	− 0.3 ± 2.2
Schröder et al. [12]	n.v.	n.v.	n.v.
Hamm [13], Michalik et al. [14]	n.v.	n.v.	n.v.

Global parameters (mean ± SD) and literature review comparison in coronal, sagittal, and transversal plane of entire group (EG) and their p -, t-values and Cohen’s d. *: p < 0.05; parameters of the younger (YG) and older group (OG) and their p values; †: Parameter not clearly assigned (compare Discussion), ‡: Uncertain value (compare Discussion) Degenhardt, et al. [10]; n = 30 women and men, 30.2 ± 9.8 years; Degenhardt, et al. [11]; n = 29 women and men, 30.1 ± 10.1 years; Schröder et al. [12], n = 89 women, 26.4 ± 4.5 years; Hamm [13] and Michalik et al. [14], n = 56, women, 23.6 ± 2.0 years

A Kolmogorow–Smirnow test confirmed the data followed a normal distribution. Independent two-sample t-tests were used to compare the YG and OG. The age groups differed in only a few parameters In CP, this held true only for the lateral flexion of L2. In SP, these were TK-angle, LL-angle, pelvic inclination, flexion of VP, T1, T12, and L1 (Tables 1, 2).

### Specific parameters

Specific parameters for the EG, and both subgroups, in all three planes are presented in Table 1.

**Fig. 2:**
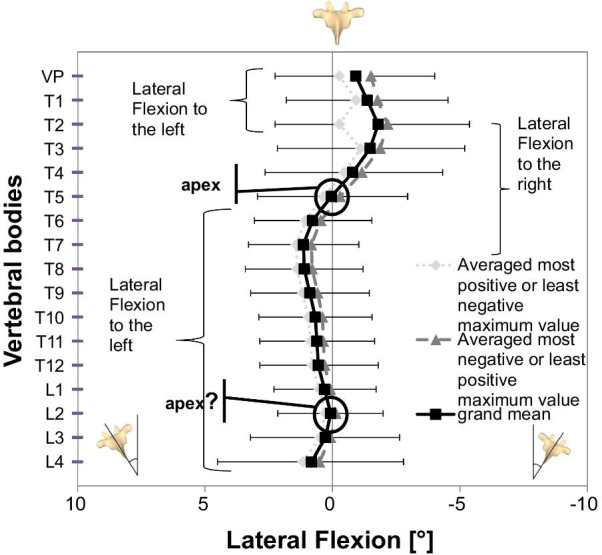
Vertebral body positions in the coronal plane. Positive values indicate tilt to the left, negative values to the right. Averaged most positive or least negative maximum value, most negative or least positive with one-sided SDs, and grand means of EG are displayed; apex, area, and direction of lateral flexion are marked

For the CP, the courses of averaged maxima curves ran in parallel; the mean body sway was small and consistent (Fig. 2). Only T2 showed an averaged deviation of the maximum positive value (least negative). Analysis revealed that the positive outliers had a larger distance to the median than the negative values, explaining this peak at T2. The vertebral bodies VP–T4 were tilted on average to the right, T6–L1 and L3–L4 were tilted to the left, while T5 and L2 were in an almost neutral position. This depicted a mean right-sided lateral flexion between T2 and T7, with the apex at T5 (displayed as point of inflection in Fig. 2). The vertebral bodies, cranial from T2 and caudal from T7, constituted a left-sided lateral flexion.

The vertebral bodies VP–T4, T6–T12, and L4 had on average a trend differing from zero with a small-to-medium effect size (Table 1).

**Fig. 3:**
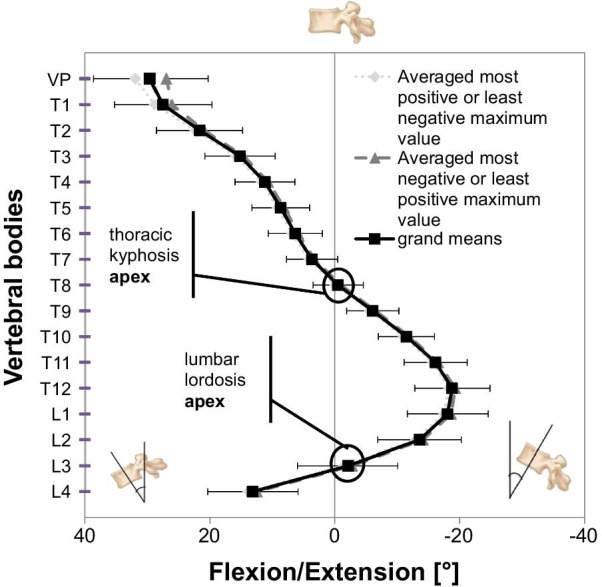
Vertebral body positions in the sagittal plane. Positive values indicate tilt toward flexion, negative values toward extension. Averaged most positive or least negative maximum value, most negative or least positive with one-sided SDs, and grand means of EG are displayed; thoracic kyphosis and lumbar lordosis apex are marked

For the SP, the averaged vertebral body positions of the EG are illustrated in Fig. 3.

**Fig. 4:**
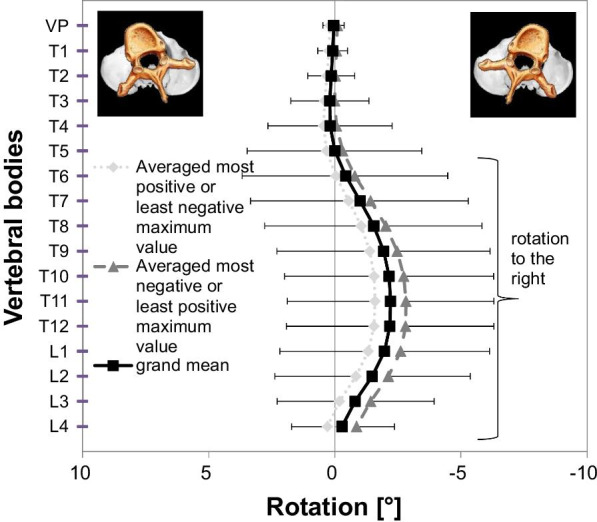
Vertebral body positions in transversal plane. Positive values mean indicate rotation to the left, negative values to the right. Averaged most positive or least negative maximum value, most negative or least positive with one-sided SDs, and grand means of EG are displayed; area and direction of rotation are marked

The courses of the averaged maxima curves ran in parallel, which indicated minimal body sway. However, the participants slightly moved their heads, as the VP and T1 (first thoracic vertebral body) show divergent courses. Error bars (scaling in Fig. 3 differs from Figs. 2 and 4) show the inter-individual posture variation as the SDs of rotation in each plane. On average, the vertebral bodies VP–T7 and L4 were tilted toward flexion, T9–L2 toward extension, while T8 and L3 are in an almost neutral position. The mean TK (VP–T12) had an apex at T8 (displayed as point of inflection in Fig. 2), and the LL (T12–L4) had an apex at L3. When comparing all parameter values’ symmetry, we found they were not symmetrical (dissimilar from zero), with the exception of T8 due to the kyphotic apex in an almost neutral position.

Within the TP, the estimated values for T6–L4 showed a rotation to the right with a small-to-medium effect size (Table 1). This is equal to a surface rotation to the left of the respective spinous process, while other vertebral bodies were in an almost neutral position (Fig. 4). Averaged maxima curves ran nearly in parallel, and there was no rotational body sway between VP and T3. From T4–L4, the swaying increased, stronger than in the CP, but was the same across all vertebrae. The rotation to the right began at T6 and had its maximum at T11 with − 2.2° ± 3.5. Interestingly, the mean 100 participants consistently showed considerable body rotation in one direction.

### Global parameters

The global parameters for the EG and both subgroups are shown for each of the three planes in Table 2, along with comparable values from other studies [10–14] in Table 2.

The results were characterized by the following values: mean trunk inclination of EG was 3.1° ± 2.1, trunk imbalance was to the left by − 0.2° ± 1.1, and the maximum surface rotation was to the left by − 3.9° ± 2.7.

## Discussion

In this current study of 100 asymptomatic healthy females, we found that specific parameters in the CP the spine were slightly tilted in both directions, while in the SP we identified the mean TK apex at T8 and the apex of LL at L3. In the TP, participants had a mean vertebral body rotation to the right (in situ vertebrae change direction of rotation). The TP maximum surface rotation was the most salient value of the global parameters and was rotated to the left across all participants. Age-specific differences were only seen in the SP and had little effect on overall posture.

### Specific parameters

In the CP, there was a mean lateral flexion to the right in the upper thoracic spine with this apex at T5. In the lower thoracic and lumbar spine, a lateral flexion to the left was seen with a nearly neutral vertebral body at L2. Although it did not seem to be an apex because L3 and L4 are not tilted in the opposite direction, the apex may be located outside the measurement area at L5 or the sacrum.

There were no unexpected results in the SP, as we expected that kyphosis would range between VP and T12 with its turning point at T8. The LL (L1–L4; L5 and sacrum were not in the measurement area) displayed an inflection point at L2, which was anticipated. However, as there are not yet studies that we can compare these findings to, we cannot verify the accuracy of these results.

In the TP, our study mean showed considerable body rotation in one direction similar to other studies [26–29] assessing spinal rotation with CT and MRI measurements, as well as analogous group results for women [26]. They found an almost identical vertebral rotation regarding the direction (right), the affected segments (T5–L3), and their extent (2.6°) [26], which was consistent with our results except that they identified a vertebral height of the maximum at T7. Furthermore, they detected that situs inversus totalis participants have vertebral rotation in the opposite direction [27]. Their maximum of − 2.7° was also at T7, showing a physiological phenomenon to have a slightly rotated thoracic spine, although it is unclear why the height of the most rotated vertebra differed in our study (T8). Though they used MRI rather than ST, this discrepancy could be attributed to the horizontal position while lying supine during the MRI. However, it was shown that there was no significant difference in the spine while standing erect or laying supine [28]. In standing position, the rotation appeared between T5 and L3, with its maximum at T7 and T8 (2.7°). Hence, the most plausible explanation resides in the dissimilar measuring methods between MRI and ST.

Taken together, body sway occurs less in the CP and SP than in TP, but values of approximately 1° should be interpreted with caution considering a measuring error of 3° for surface rotation when compared to radiography [30]. In static [9] and dynamic [5] measurements (apparative model examination: average deviation of approx. 150 mm), validity and reliability were shown. Measurement differences between the motion analytical gold standard (VICON) and the applied surface topography are 0.1–1.1% [31]. To our knowledge, this is the first study to describe the specific vertebral body positions using ST measurements. Therefore, the interpretation of the data may be challenging in some circumstances, such as the lateral flexion (CP), the exact description of the apex, and inflection points of the spinal curvatures.

### Global parameters

In the CP, the mean trunk values of the EG were negative, which meant that the trunk was slightly tilted to the left (− 1.9 mm ± 8.9). The results of a different group [10, 11] were positive, but regarding the comparable relatively high SDs (1.0 mm ± 7.2, 1.3 mm ± 5.6), the differences diminish. However, further research is warranted to examine whether groups differ by handedness, or other parameters, that could explain the high SD. For the EG, the pelvis exhibited an almost neutral position consistent with other research [10, 13], while only one study showed a relevant imbalance accounting for their high trunk deviation values. The maximum apical deviation to the left (EG: − 5.1° ± 3.7) was similar to previously published values (− 5.0° ± 4.1) [10]. Comparison of the values to the right was compromised since they were not reported consistently [10]. There are no comparisons currently available for shoulder obliquity, as this is a new parameter not previously described.

The EG data in the SP were similar to that reported in other studies, especially when referencing the high SDs [10–13], although there were some marked observed differences. The TK and LL angles were on average higher in the OG, whereas the pelvic inclination angle (°) was smaller. The mean TK-angle (°) (EG) was comparable in all studies, apart from one with a lower value likely due to differing parameters (VP–T12) [13, 14], as well as the LL-angle (°) (T12–DM) [13, 14]. Differing values could also be attributed to different distributions in the respective study groups. One study described that females experience an increase in LL during the 20–30 s [32], which could account for these lower values as they were on average younger than our YG. The lower LL of that study [10] may be explained by the inclusion of men, as men have significantly lower LL values than women [12, 32]. However, the literature also notes that LL decreases with age (≥ 40), contradictory to the results we present in the current study [32, 33]. The pelvic inclination (°) mean value of our YG was similar to other data [12]. Unfortunately, the authors did not include how the pelvic inclination was measured [12], i.e., dimples’ pelvic inclination or the symmetry lines. Already reported slightly smaller values [10] could also be explained by the inclusion of men in the sample, as they have smaller LL-angels that affect the pelvic inclination.

Only a few studies comment on parameters described in the TP. The EG mean maximum surface rotation to the left is − 3.9° ± 2.7 and 2.0° ± 2.4 to the right. Similar to the CP, a higher EG value of the maximal surface rotation was negative denoting a rotation to the left. In contrast, other available highest value [10, 11] indicated a rotation to the right, but with the same limitations of inconsistent reporting (10).

Currently, orthopedists and physiotherapists work with the hypothesis that a normal healthy spine is straight and symmetrical, but these results challenge this assumption and suggest further research in this area should be considered.

Potential limitations arise from the usage of additional markers, as they were necessary to analyze the gait patterns of the framework project. Furthermore, C7 and both SIPS were marked by palpation. However, both of these limitations may only slightly contribute to the deviations for the estimated values. Additionally, the visual fixation, as participants were not allowed to wear shoes, and a non-standardized habitual standing position during the measurements could have influenced the results. In addition, it must be noted that although the asymptomatic subjects did not show any functional abnormalities, it was not checked by means of X-ray or MRI scans whether, for example, small deformities existed. Due to the large number of hypothesis testing, significant values should be interpreted with caution and indicated for trends, which can be seen as a further limitation of the work.

## Conclusions

Displayed by large SDs, we found a high variation in posture for asymptomatic healthy female volunteers. Moreover, it was shown that asymmetrical postures were also common in healthy women. In the CP, the upper thoracic spine was tilted to the right lateral flexion, while to the left in the lower thoracic spine. Within the SP, the thoracic apex resided at T8 and the lumbar apex at L3. The asymmetrical posture was mostly characterized by the findings in the TP, with a mean vertebral body rotation to the right between T6 and L4. Women’s age differences were predominantly seen in the SP, effecting TK-angle, LL-angle, pelvic inclination, flexion of T1, T12, and L1. This dataset may be used as normative reference values to compare similar measurements in medical practices. The physiological asymmetrical posture of women should be considered when assessing spinal posture. With regard to spinal surgery, it should be clarified whether an approximation to an absolutely symmetrical posture is desirable from a biomechanical point of view. Further studies are warranted to establish a normative dataset for men, as well as studies that investigate patients with back pain, situs inversus totalis, and arthrosis of lower extremities to assess the influences of these and other pathologies on posture.

## Supplementary Information

**Additional file 1.** Definition and images of selected parameters. This document explains the selected global and specific parameters in more detail. Both with the help of graphical illustrations and by definition.

## Data Availability

All results of data analyses pertaining to this study are included in this published article. The datasets generated during the current study are not publicly available since they are a part of a future doctoral thesis. They will be made available by the corresponding author on reasonable request.

## Abbreviations

C: Cervical vertebral body
CP: Coronal plane
DL: Dimple left
DM: Dimple midpoint
DR: Dimple right
EG: Entire group
ICT: Inflection point between cervical and thoracic spine
ILS: Inflection point between lumbar spine and sacrum
ITL: Inflection point between thoracic and lumbar spine
L: Lumbar vertebral body
LL: Lumbar lordosis
OG: Older group
SD: Standard deviation
SP: Sagittal plane
ST: Surface topography
T: Thoracic vertebral body
TK: Thoracic kyphosis
TP: Transverse/transversal plane
VP: Vertebra prominens
YG: Younger group
