# Supplementary material for: Evaluation of 3D vertebral and pelvic position by surface topography in asymptomatic females: presentation of normative reference data
Source: J Orthop Surg Res. 2021 Dec 4;16:703. doi: 10.1186/s13018-021-02843-2 (PMC8642978; doi:10.1186/s13018-021-02843-2)
Supplement: Supplementary file 1 — Additional file 1. Definition and images of selected parameters. This document explains the selected global and specific parameters in more detail. Both with the help of graphical illustrations and by definition. [file 13018_2021_2843_MOESM1_ESM.pdf]

Evaluation of 3D Vertebral and Pelvic Position by Surface Topography in Asymptomatic Females: Presentation of normative reference data

Definition and images of selected parameters

**Parameters**	**Definitions**
	**Sagittal plane**
Trunk Inclination VP - DM [mm], [°] 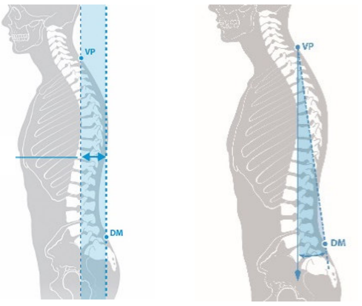	The trunk inclination refers to a difference in distance between two plumb lines dropped from VP (Vertebra Prominens) and DM (Dimple Midpoint), based on a vertical plane (sagittal section) (in mm) or rather is defined by the angle between the plumb line dropped from VP and the line between VP and DM (in degrees). A positive value means VP is more ventral than DM.
Thoracic Kyphosis Angle [°] 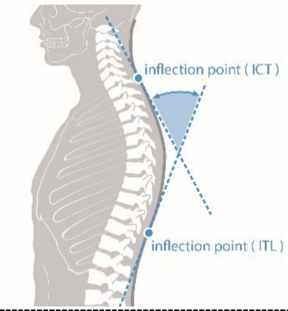	The thoracic kyphosis is calculated by the angle formed between the two surface tangent lines of the ICT (inflection point between cervical and thoracic spine) and ITL (inflection point between thoracic and lumbar spine), based on the spinous process line.
Lumbar Lordosis Angle [°] 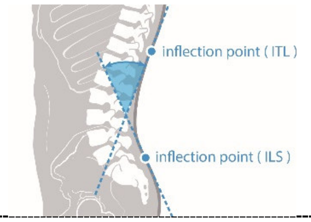	The lumbar lordosis is calculated by the angle formed between the two surface tangent lines of the ITL (inflection point between thoracic and lumbar spine) and ILS (inflection point between lumbar spine and sacrum), based on the spinous process line.

Pelvic Inclination (dimples) [°]<br>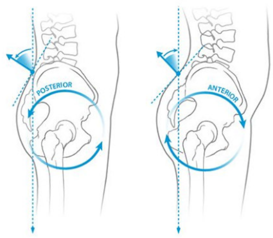	The pelvic inclination (dimples) is the mean vertical torsion of the two surface normals on the left and right dimple (DL and DR).<br>A positive value signifies a mean vertical component upwards (anterior tilt).
Vertebral sagittal Flexion and Extension [°]<br>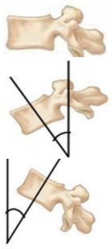	The parameter describes the inclination of the calculated vertebra in space (relative to a plumb / gravity line) as seen from a left view. The angle (in degrees) is calculated from the projection of the vertebra in a sagittal plane (rotation and lateral flexion are ignored).<br>A positive value means a forward tilt of the vertebra (flexion).
	**Coronal plane**
Trunk Imbalance VP - DM [mm], [°]<br>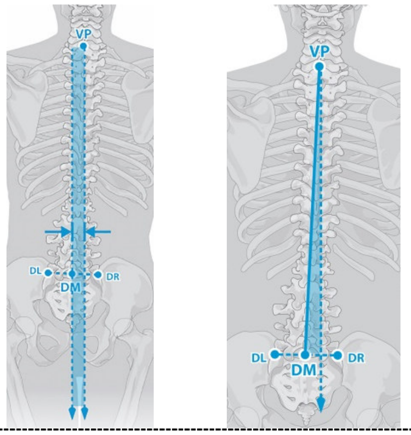	The trunk imbalance in the coronal plane is defined by the difference of the lateral distance between VP and DM, based on a coronal plane (in mm) or rather the angle between the plumb line dropped from VP and the line between VP and DM (in degrees).<br>A positive value means that the patient is leaning to the right.
Maximum Apical Deviation [mm]<br>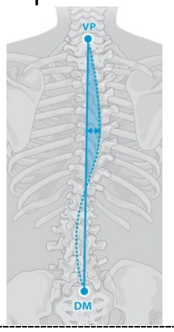	The maximum apical deviation is measured from the apex of the largest curve – either left or right – in the coronal plane to the line drawn between VP and the DM.<br>A positive value means a deviation to the right.

Shoulder Obliquity [mm], [°]<br>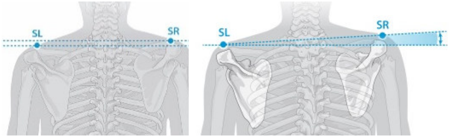	The shoulder obliquity is the difference (in mm) between two horizontal lines drawn through both acromia. The acromion is identified on each shoulder (SR: shoulder right and SL: shoulder left).<br>A line between those two points is drawn and compared with the horizontal line representing the horizon (perpendicular to the gravity line). The angle (in degrees) between them is measured.<br>A positive value means that the right shoulder is elevated.
Pelvic Obliquity [mm], [°]<br>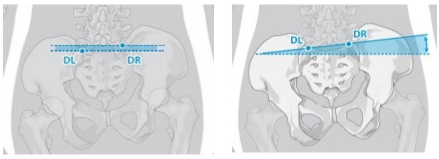	The pelvic obliquity is the difference (in mm) between two horizontal lines drawn through both dimples (DL and DR).<br>A line is drawn from DL to DR and is compared to a horizontal line representing the horizon. The angle (in degrees) between them is measured.<br>A positive value means that the right pelvis is elevated.
Vertebral Lateral Flexion [°]<br>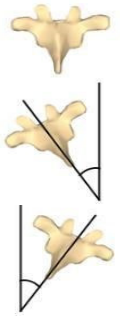	The parameter describes the lateral inclination of the vertebra in space (relative to a plumb / gravity line) as seen from a posterior-anterior view. The angle (in degrees) is calculated from the projection of the vertebra in the coronal plane (rotation and sagittal extension / flexion are ignored).<br>A positive value means a tilt of the vertebra to the left (lateral flexion left).
	**Transversal plane**
Maximum Surface Rotation [°]<br>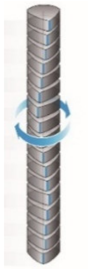	The maximum surface rotation in the transversal plane is defined by the rotation in the axial plane of a spinous process when compared to the neutral pelvis (left dimple - right dimple line in parallel to the coronal plane) is recorded in degrees.<br>A positive value means a maximum rotation to the right.

Pelvic Rotation [°]<br>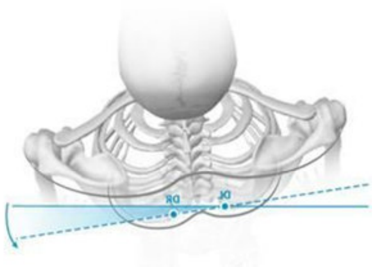	The pelvic rotation is the rotation in the transversal plane of the right dimple relative to a reference coronal plane that is defined from the system setup, perpendicular to the camera-projection axis.<br>A positive value means the pelvis is rotated to the left when seen from behind (the value is corrected * (-1)).<br><br>All 3D-modells and rotation values of the 12 frames are normed according to the original pelvic rotation value of the selected average frame. Therefore, the whole body is rotated until the pelvis is in a neutral position (left dimple - right dimple line in parallel to the coronal plane); afterwards all other parameters are calculated.
Vertebral Rotation [°]<br>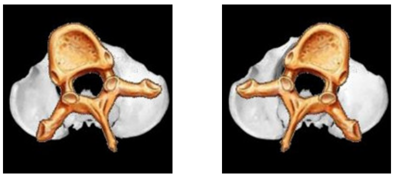	The vertebral rotation describes the rotation of a vertebra in the transversal plane (relative to the neutral pelvis).<br>A positive value means a vertebra is rotated to the left (counterclockwise) when seen from behind.<br><br>The rotation of vertebral bodies happens in situ, therefore the direction of rotation between surface and vertebral rotation changes. Hence, a surface rotation to the right, mathematically represented with a +, becomes a vertebral body rotation to the left. This is due to the calculation process in which a vector is used that points from Processus spinosus towards the middle of the vertebral body meaning that the surface rotation changes its direction within the vertebral body.

Definitions and images were assigned by DIERS (© Florian Franke / DIERS) and adapted by Claudia Wolf.
